# Relation between vitamin D deficiency and diabetic maculopathy

**DOI:** 10.1038/s41598-025-08941-z

**Published:** 2025-07-04

**Authors:** Ahmed A. Khater, Mohammed N. Elmohamady, Tarek Ibrahim Badr, Mohamed Moustafa Baz, Ahmed Sherin M. Bayoumy

**Affiliations:** 1 Ophthalmology Department, Faculty of Medicine, Benha University, Police st.,Shebin al-Qanater, Benha, Qaliopia 13711 Egypt; 2 Memorial Institute for Ophthalmic Research (MIOR), Giza, Egypt; 3 Ophthalmology Department, Faculty of Medicine, Benha University, 79 Nozha st Heliopolis, Cairo, Egypt

**Keywords:** Vitamin D, Diabetic, Macular edema, Ischemia, OCTA, Diseases, Health care, Medical research, Risk factors

## Abstract

Vitamin D deficiency has been linked to DR progression, its specific role in diabetic maculopathy remains underexplored. This study aimed to evaluate the relationship between serum vitamin D levels and diabetic maculopathy. In this cross-sectional study, 68 patients with diabetic macular edema (DME) underwent comprehensive ophthalmic examinations, including OCTA to assess superficial and deep vascular density and foveal avascular zone (FAZ), alongside measurement of serum vitamin D levels. Patients with renal impairment, granulomatous diseases, or vitamin D supplementation were excluded. Of the 68 patients (54.4% female, 45.6% male; mean age 58 ± 7 years), vitamin D levels showed significant positive correlations with superficial vascular density in foveal (*r* = 0.711, *P* < 0.001), parafoveal (*r* = 0.852, *P* < 0.001), and perifoveal zones (*r* = 0.832, *P* < 0.001), and with deep vascular density in foveal (*r* = 0.868, *P* < 0.001), parafoveal (*r* = 0.790, *P* < 0.001), and perifoveal zones (*r* = 0.645, *P* < 0.001). Negative correlations were observed with FAZ in superficial (*r* = − 0.806, *P* < 0.001) and deep layers (*r* = − 0.801, *P* < 0.001). Multivariate linear regression, controlling for age, gender, and diabetes duration, confirmed vitamin D as a significant predictor of superficial and deep vascular density and FAZ parameters. Diabetic macular ischemia is closely linked to vitamin D deficiency, as shown by reduced vascular density and enlarged FAZ. These findings suggest that vitamin D may help prevent retinal microvascular damage in diabetic retinopathy.

## Introduction

Diabetic macular edema (DME) is main cause of visual loss in diabetic retinopathy (DR) and can occur in the different stages of DR. DME results mainly from retinal micro-vascular changes disturbing the blood-retinal barrier^1^. Diabetic macular ischemia (DMI) is considered an irreversible diabetic maculopathy, and its presence limits the benefits of potential treatments for DR^2,3^. It is characterized by irregularly enlarged foveal avascular zone (FAZ), and macular capillary dropout^4,5^. Several risk factors, including hyperglycaemia, hypertension, dyslipidaemia, and longer diabetes duration, are involved in the pathophysiology of DR^6,7^. Proper control of blood glucose, blood pressure, and lipid levels has been shown to delay the onset and progression of DR by reducing microvascular damage and inflammation^6,8,9^.

The development of optical coherence tomography (OCT) has allowed imaging of DME with high resolution tomography^10^. Also, optical coherence tomography angiography (OCTA) has been widely utilized to quantify the macular perfusion density of the deep retinal capillary plexus (DCP), the superficial retinal capillary plexus (SCP) and the foveal avascular zone (FAZ) parameters^11–13^.

Vitamin D is important for a variety of physiologic processes and its deficiency has become a worldwide problem^14^. Vitamin D deficiency has been implicated in various diseases, including diabetes mellitus, hypertension, and cardiovascular disease^15–17^. Vitamin D exerts both anti-inflammatory and anti-angiogenic effects^14^. Vitamin D receptors are expressed extensively in the retina^18^ and in experimental models vitamin D closely regulates Vascular Endothelial Growth Factor (VEGF)^19^.The active form of vitamin D was found to be a potent inhibitor of retinal neovascularization^20^, and could decrease levels of VEGF^21,22^. Through several mechanisms vitamin D deficiency seems to play a role in the development and progression of diabetic retinopathy^23,24^.

While prior studies have explored the association between serum vitamin D levels and DR, including PDR and DME^25–29^, the relationship with DMI remains poorly understood. DMI, characterized by capillary dropout and enlarged FAZ, is a severe and irreversible form of diabetic maculopathy with limited treatment options^3–5^. Experimental studies suggest that vitamin D’s anti-angiogenic and anti-inflammatory effects may protect against retinal microvascular damage^19–22^, but no studies have specifically investigated its role in DMI. This study aims to address this gap by evaluating the relationship between serum vitamin D levels and diabetic maculopathy, with a focus on DMI parameters assessed via OCTA.

## Methodology

We designed a cross-sectional study that was approved by the Institutional Review Board of the Memorial Institute of Ophthalmic Research, Giza (MIOR 0017) and met the Declaration of Helsinki. Informed consent was obtained from all patients to participate in the study. The study was conducted in the Memorial Institute of Ophthalmic Research, Giza, Egypt and Ophthalmology Department, Benha University Hospitals, Benha, Egypt.

### Study subjects

Subjects with clinical diabetic maculopathy were considered for inclusion while patients with the following conditions were excluded : renal impairment (eGFR < 30 mL/min/1.73 m2(CKD stages 4 and 5)), granulomatous diseases (tuberculosis, sarcoidosis, etc.), and malabsorption syndromes (coeliac disease, bacterial overgrowth, and concomitant orlistat treatment), use of drugs affecting serum vitamin D levels (e.g., anticonvulsants, and glucocorticoids), pregnant and lactating women, chorio-retinal disorder other than DR and DME (e.g., retinal vascular occlusion, and age-related macular degeneration), Subjects that presented motion artifacts during OCTA or poor signal strength, history of intravitreal injections or intraocular surgery except uncomplicated cataract surgery and those currently on vitamin D supplementation.

### Study examination

All subjects had complete ophthalmic examination including corrected visual acuity, slit lamp biomicroscopic examination, and dilated fundus examination. Patients found to have clinical diabetic macular oedema underwent further assessment by OCTA to confirm diagnosis and classify DME. Laboratory assessment of vitamin D level was done.

DME was diagnosed based on clinical examination (hard exudates, retinal thickening within 2-disc diameters of the fovea) and confirmed by OCT showing intraretinal or subretinal fluid, cystoid spaces, or increased central macular thickness (> 300 μm). DMI was identified via OCTA, characterized by an enlarged foveal avascular zone (FAZ > 500 μm^2^) or reduced vascular density in the superficial or deep capillary plexus (< 40% in foveal regions). Diabetic retinopathy was staged according to the Early Treatment Diabetic Retinopathy Study (ETDRS) classification, based on fundus examination and photography. Patients with mild, moderate, or severe non-proliferative diabetic retinopathy (NPDR) or proliferative diabetic retinopathy (PDR) were included, provided they had clinical diabetic maculopathy.

OCTA was conducted using the Optovue RTVue XR Avanti (Optovue, Inc., Fremont, CA, USA), capturing a 6 × 6 mm scanning area centered on the fovea to evaluate macular thickness, superficial and deep vascular density, and FAZ. A built-in OCTA software automatically segments the superficial capillary plexus (SCP) and deep capillary plexus (DCP), utilizing a split-spectrum amplitude-decorrelation angiography algorithm. The RTVue XR Avanti performs 70,000 A-scans per second, acquiring two consecutive B-scans at 304 locations with motion correction technology to minimize artifacts, completing the scan in approximately 3 s. Poor signal strength was defined as a signal strength index (SSI) below 50, indicating suboptimal image quality for accurate vascular density or FAZ measurements. The macular regions were delineated using the ETDRS grid on the 6 × 6 mm scan: the foveal region as the central 1 mm diameter circle, the parafoveal region as the annulus between 1 and 3 mm, and the perifoveal region as the annulus between 3 and 6 mm.

### Statistical analysis

Data management and statistical analysis were performed using SPSS version 28 (IBM, Armonk, New York, United States). Quantitative data was assessed for normality using the Kolmogorov–Smirnov test and direct data visualization methods. According to normality, quantitative data was summarized as means and standard deviations or medians and ranges. Categorical data was summarized as numbers and percentages. Correlations between vitamin D_3_ and other parameters were executed using Pearson’s or Spearman’s correlation. DR stage was included as a covariate in multivariate regression analyses to control its potential confounding effect on vitamin D and OCTA parameters. Multivariate linear regression analyses were done to assess vitamin D_3_ as a predictor of different OCTA parameters. The regression coefficients with 95% confidence intervals were calculated. All statistical tests were two-sided. P values less than 0.05 were considered significant.

## Results

### Demographics

The average age was 58 years (SD ± 7). The gender distribution was balanced, with 31 males (45.6%) and 37 females (54.4%). The duration of diabetes mellitus (DM) among the participants varied, with a median duration of 11 years, ranging from 5 to 25 years. The average glycated hemoglobin (HbA1C) level was found to be 7.9% (SD ± 0.8). Additionally, the mean serum vitamin D_3_ level was 23.9 ng/mL (SD ± 3.8) **(**Table 1**).** Also, we reported the distribution of DR stages (of the 68 participants, 40% had moderate NPDR, 30% had severe NPDR, and 30% had PDR).

**Table 1:** Demographic characteristics of the studied patients.

General characteristics		
Age (years)	Mean ± SD	58 ± 7
**Gender**		
Males	n (%)	31 (45.6)
Females	n (%)	37 (54.4)
DM duration (years)	Median (range)	11 (5–25)
HbA1C	Mean ± SD	7.9 ± 0.8
Vitamin D_3_ (ng/mL)	Mean ± SD	23.9 ± 3.8

### OCTA

The mean foveal retinal thickness was 334 μm (SD ± 43), while the parafoveal and perifoveal thicknesses were slightly higher, measured at 376 μm (SD ± 47) and 359 μm (SD ± 44), respectively. In terms of superficial vascular density, the foveal region showed a lower density of 22.6% (SD ± 4.3) compared to the parafoveal and perifoveal regions, which had densities of 49.5% (SD ± 8.3) and 48.4% (SD ± 7.8), respectively. The deep vascular density followed a similar pattern, with the foveal region having a density of 36.5% (SD ± 6.1), while the parafoveal and perifoveal densities were 52.3% (SD ± 6) and 48.9% (SD ± 8.2), respectively. Additionally, measurements of the foveal avascular zone (FAZ) showed significant variation in different layers: the auto FAZ measured 434 µm^2^ (SD ± 42), the superficial layer measured 502 μm (SD ± 49), and the deep layer was the largest, measuring 584 μm (SD ± 64) (Table 2).

**Table 2:** OCTA parameters in the studied patients.

	Mean ± SD
Retinal thickness (um)	
Foveal	334 ± 43
Parafoveal	376 ± 47
Perifoveal	359 ± 44
**Superficial vascular density (%)**	
Foveal	22.6 ± 4.3
Parafoveal	49.5 ± 8.3
Perifoveal	48.4 ± 7.8
**Deep vascular density (%)**	
Foveal	36.5 ± 6.1
Parafoveal	52.3 ± 6
Perifoveal	49.3 ± 6.4
**FAZ (um** ^**2**^ **)**	
Auto	434 ± 42
Superior	502 ± 49
Deep	584 ± 64

*FAZ* Foveal avascular zone, *SD* Standard deviation.

### Correlation between vitamin D_3_ and other parameters

**Fig. 1:**
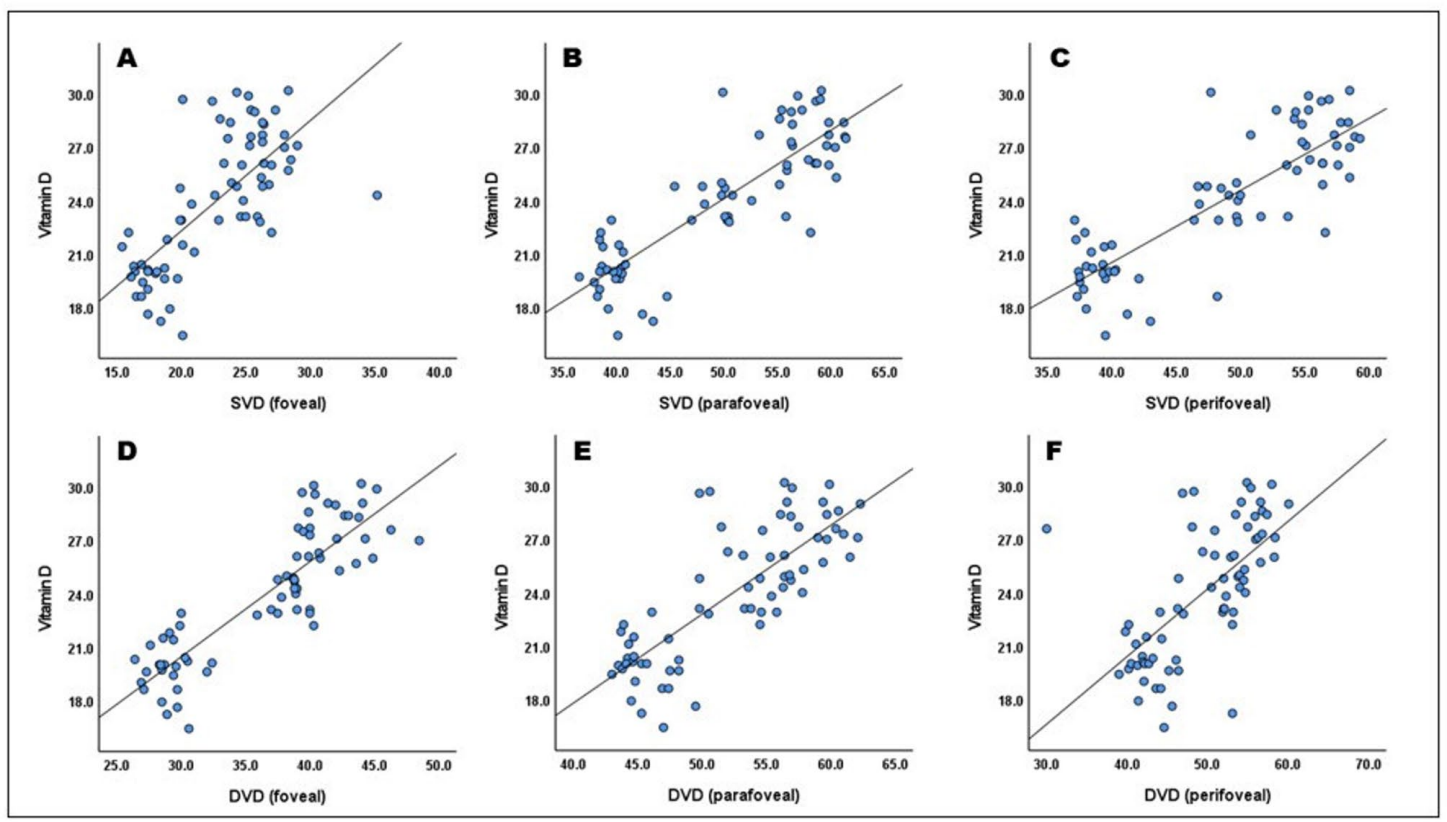
Correlation between vitamin D and (**a**) SVD-foveal; (**b**) SVD-parafoveal; (**c**) SVD-perifoveal; (**d**) DVD-foveal; (**e**) DVD-parafoveal; (**f**) DVD-perifoveal.

Vitamin D_3_ showed a significant positive correlation with superficial vascular density in the foveal (*r* = 0.711, *P* < 0.001), parafoveal (*r* = 0.852, *P* < 0.001), and perifoveal zones (*r* = 0.832, *P* < 0.001). Additionally, vitamin D was significantly positively correlated with deep vascular density in foveal (*r* = 0.868, *P* < 0.001), parafoveal (*r* = 0.790, *P* < 0.001), and perifoveal zones (*r* = 0.645, *P* < 0.001) (Table 3; Fig. 1).

**Table 3:** Correlation between vitamin D and other parameters.

	Vitamin D	
	*r*	*P*
DM duration (years)	− 0.526	< 0.001*
HbA1C	− 0.138	0.26
Retinal thickness		
Foveal	− 0.157	0.202
Parafoveal	− 0.138	0.262
Perifoveal	− 0.138	0.262
Superficial vascular density		
Foveal	0.711	< 0.001*
Parafoveal	0.852	< 0.001*
Perifoveal	0.832	< 0.001*
Deep vascular density		
Foveal	0.868	< 0.001*
Parafoveal	0.790	< 0.001*
Perifoveal	0.645	< 0.001*
FAZ		
Auto	− 0.896	< 0.001*
Superficial	− 0.806	< 0.001*
Deep	− 0.801	< 0.001*

* Significant P-value; *FAZ* Foveal avascular zone.

**Fig. 2:**
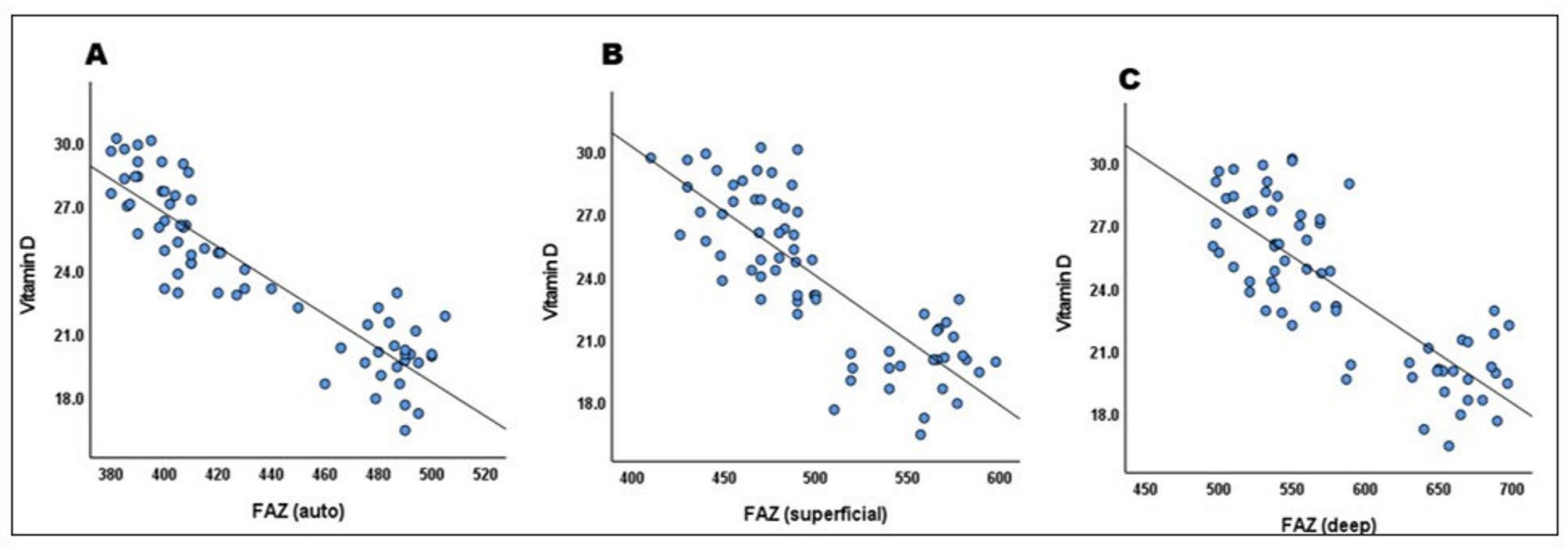
Correlation between vitamin D and (**a**) FAZ-auto; (**b**) FAZ-superficial; (**c**) FAZ-deep.

In contrast, vitamin D_3_ revealed significant negative correlations with DM duration (= 0.525, *P* < 0.001) and FAZ auto (*r* = − 0.896, *P* < 0.001), superficial (*r* = − 0.806, *P* < 0.001), and deep (*r* = − 0.801, *P* < 0.001) (Table 3; Fig. 2).

No significant correlations were observed between vitamin D_3_ with HBA1C (*P* = 0.260) and retinal thickness in foveal (*P* = 0.202), parafoveal (*P* = 0.262), and perifoveal (*P* = 0.262) zones (Table 3).

### Prediction of OCTA parameters using vitamin D

Multivariate linear regression analyses were performed to assess vitamin D_3_ as a predictor of retinal thickness, superficial vascular density, deep vascular density, and FAZ, controlling for age, gender, and DM duration (Table 4).

**Table 4:** Multivariate linear regression analysis for vitamin D to predict OCTA parameters.

	B (95% CI)^†^	*P*-value
Retinal thickness		
Foveal	− 2.299 (− 6.591–1.993)	0.289
Parafoveal	− 2.101 (− 6.788–2.585)	0.374
Perifoveal	− 1.845 (− 6.251–2.56)	0.406
Superficial vascular density		
Foveal	1.059 (0.772–1.346)	< 0.001*
Parafoveal	2.001 (1.569–2.434)	< 0.001*
Perifoveal	1.84 (1.417–2.264)	< 0.001*
Deep vascular density		
Foveal	1.552 (1.261–1.843)	< 0.001*
Parafoveal	1.339 (0.975–1.703)	< 0.001*
Perifoveal	1.255 (0.766–1.744)	< 0.001*
FAZ		
Auto	− 10.605 (− 12.484 to − 8.727)	< 0.001*
Superior	− 11.707 (− 14.606 to − 8.807)	< 0.001*
Deep	− 15.557 (− 19.324 to − 11.789)	< 0.001*

* Significant P-value; †Adjusted for age, gender, DM duration; B: Regression coefficient; 95% CI: 95% Confidence interval; FAZ: Foveal avascular zone.

For retinal thickness, vitamin D_3_ was not a significant predictor in the foveal, parafoveal, and perifoveal zones (Table 4).

Vitamin D was a significant predictor for superficial vascular density in the foveal (B = 1.059, 95% CI = 0.772–1.346, *P* < 0.001), parafoveal (B = 2.001, 95% CI = 1.569–2.434, *P* < 0.001), and perifoveal zones (B = 1.840, 95% CI = 1.417–2.264, *P* < 0.001), controlling for age, sex, and DM duration (Table 4).

Additionally, vitamin D was a significant predictor for deep vascular density in the foveal (B = 1.552, 95% CI = 1.261–1.843, *P* < 0.001), parafoveal (B = 1.339, 95% CI = 0.975–1.703, *P* < 0.001), and perifoveal zones (B = 1.255, 95% CI = 0.766–1.744, *P* < 0.001), controlling for age, sex, and DM duration (Table 4).

For the FAZ, vitamin D was a significant predictor of FAZ auto (B = − 10.605, 95% CI = − 12.484 to − 8.727, *P* < 0.001), FAZ superficial (B = − 11.707, 95% CI = − 14.606 to − 8.807, *P* < 0.001), and FAZ deep (B = − 15.557, 95% CI = − 19.324 to − 11.789, *P* < 0.00), controlling for age, sex, and DM duration (Table 4).

## Discussion

Vitamin D deficiency is a common health problem all over the world. Although in the Middle East we have abundant sunshine, yet cultural practices and lifestyle choices lead to high rates of vitamin D deficiency through limitation of sun exposure and absence of dietary vitamin D supplementation^30,31^. Vitamin D is a known antioxidant and anti-inflammatory, thus playing a crucial role in vascular health and retinal function. Vitamin D deficiency could exacerbate inflammatory process linked to diabetic retinopathy and subsequent development of diabetic macular edema^32^.

Vitamin D deficiency has been linked to various risk factors for diabetic retinopathy including difficult glycemic control from insulin resistance and beta cell dysfunction, hypertension and cardiovascular disease^15–17^.Also a positive correlation was postulated between vitamin D deficiency and retinopathy in experimental studies^33^.

Vitamin D deficiency may contribute to diabetic macular ischemia through several mechanisms. First, vitamin D is a potent regulator of vascular endothelial growth factor (VEGF), a key mediator of retinal neovascularization and vascular permeability and can inhibit VEGF via downregulation of hypoxia-inducible factor-1α (HIF-1α)^19–21^. Low vitamin D levels may lead to unchecked VEGF activity, exacerbating microvascular damage and capillary dropout, which are hallmarks of DMI. Second, vitamin D has a role in maintaining retinal vascular integrity and can affect endothelial function as it modulates nitric oxide (NO) bioavailability and reduces oxidative stress, preserving microvascular integrity^17,18,32^. Deficiency accelerates endothelial apoptosis, contributing to capillary non-perfusion. Third, vitamin D’s anti-inflammatory properties help modulate retinal inflammation, a critical driver of DR progression as it may suppress pro-inflammatory cytokines (e.g., IL-6, TNF-α) that promote leukostasis and retinal ischemia^14^. Deficiency may intensify inflammatory cascades, leading to endothelial dysfunction and ischemia.

From its debut in 1961, FA has been the gold standard imaging modality. It does, however, require venipuncture, and although rare, incidents of allergy and mortality associated with contrast injections have been described^34,35^. Furthermore, the method is expensive and time-consuming, with a 10-minute frame acquisition time^36–38^. Although there have been conflicting findings, OCTA has become a viable substitute for identifying retinal non perfusion in diabetic patients. The noninvasive DMI detection offered by OCTA is one of its main advantages over FFA^39–41^. As a result, DMI can be detected in the early course of the disease with little or no symptoms.

An inverse relationship between serum vitamin D level and DR severity was concluded by some previous studies^42–45^ and not proven by others^28,46^.One meta-analysis reviewed vitamin D level in 4970 patients with PDR and NPDR and concluded that the risk for PDR increased by 60% in patients with Vitamin D deficiency^27^. Another meta-analysis Searching published studies on midline and EMBASE till July 2015 found a significant correlation between Vitamin D deficiency and DR with a statistically difference between DR and non-DR patients in serum Vitamin D levels^25^.Interestingly demographic studies on certain populations was also carried out, we reviewed a study carried on 3226 Asian Indian patients confirming association between severity of vitamin D deficiency and that of DR^47^.

Our work revealed no significant correlation between vitamin D deficiency and DME in terms of macular thickness and this compares with previous studies^28,29,48^. Our retinal thickness readings in foveal, parafoveal and perifoveal regions were not correlated to Vitamin D deficiency (*r*=−0.157, −0.138, −0.138 respectively with *p* < 0.001 in each). Our main finding was the negative correlation between serum vitamin D level and DMI in form of significant negative correlation between serum vitamin D level and FAZ proven by the increase in the FAZ area at both superficial and deep levels (*r*=−0.806, −0.801 respectively with *p* < 0.001) and significant positive correlation between it and superficial and deep vascular density in foveal, parafoveal and perifoveal regions with *p* < 0.001 in each as shown in Table 3. Multivariate linear regression analyses were performed to exclude possible confounders as age and DM duration and confirmed the significant correlation between vitamin D deficiency and DMI as shown in Table 4.

Despite previous reports studying the relation between vitamin D level and diabetic retinopathy including PDR, its relation to DMI was not clearly described^25,42,46,49^.Also we observed no significant correlation with HBA1C (*P* = 0.260) as Bonakdaran et al. suggested^46^. Bonakdaran et al.^46^ found no significant difference in serum vitamin D level among patients with or without DR. Additionally, no significant correlation was found between vitamin D level and other known risk factors of DR, including poor glycemic control, diabetes duration, hypertension, inflammation, and insulin growth factor.

In conclusion we believe that Vitamin D deficiency is strongly correlated to DMI, and unfortunately there are no guidelines for Vitamin D supplementation for patients with diabetic retinopathy, as maintaining adequate Vitamin D levels could mitigate diabetic maculopathy. Further research using large case control groups, comparing serum and aqueous vitamin D levels with the results, including intraocular levels of VEGF or other inflammatory cytokines related to DME, and correlating vitamin D levels with severity of macular ischemia is recommended. Also, Prospective studies for treatment of diabetic maculopathy patients with Vitamin D supplementation together with adequate glycemic control will be conclusive for the role of vitamin D in treatment of diabetic maculopathy.

## Data Availability

The data sets used and/or analyzed during the current study are available from the corresponding author on reasonable request.
